# Environment‐induced heat stress causes ventricular‐dependent biochemical changes in the heart in female pigs

**DOI:** 10.14814/phy2.70414

**Published:** 2025-06-07

**Authors:** Melissa Roths, Tori E. Rudolph, Alyssa D. Freestone, Lance H. Baumgard, Joshua T. Selsby

**Affiliations:** ^1^ Department of Animal Science Iowa State University Ames Iowa USA

**Keywords:** heat stroke, heatwave, hyperthermia, muscle, proteolysis

## Abstract

Prolonged exposure to inescapable heat and humidity can lead to environment‐induced heat stress (EIHS). The extent to which EIHS damages the heart is largely unknown, though our previous work indicated EIHS caused ventricle‐dependent changes. The purpose of this investigation was to determine the extent to which EIHS increased proteolysis and altered calcium homeostasis in the left (LV) and right ventricles (RV). We hypothesized that in the RV, EIHS would increase proteolysis, whereas in the LV, EIHS would cause calcium dysregulation. To test this hypothesis, 3‐month‐old female pigs were assigned to thermoneutral (TN; 20 ± 0.2°C; *n* = 8) or EIHS (37.4 ± 0.2°C; *n* = 8) conditions for 24 h and hearts were removed. In the RV, we discovered increased markers of proteolysis such that the relative protein abundance of calpain II, MuRF‐1, and MAFbx/Atrogin1 was increased, as was a marker of calpain activity. Conversely, in the LV, we discovered that EIHS increased the relative protein abundance of calcium regulatory proteins, including PMCA, SERCA2a, STIM1, calsequestrin, CaMKII, and VDAC. These data demonstrate EIHS caused ventricular‐dependent changes such that in the RV, the balance of proteostasis was shifted toward proteolysis and in the LV, calcium dysregulation may underlie, at least in part, our previous discovery of ventricular thickening.

## 1 INTRODUCTION

Climate change is a continuing and expanding threat to human health due, in part, to the increased frequency and duration of hot environmental conditions (Crowley & Physicians HaPPCotACo, 2016). Prolonged exposure to inescapable heat and humidity can lead to environment‐induced heat stress (EIHS), where the environmental conditions limit effective heat transfer from the body to the environment. While these environmental conditions will undoubtedly increase the frequency of heat stroke, which is generally associated with neurological involvement, they will also increase the frequency of EIHS, which is far more common (Abdelmoety et al., 2018; Harduar Morano & Watkins, 2017). Additionally, outdoor workers, the elderly, people without the logistical or economic means to support artificial cooling, and individuals with impaired thermoregulation or cardiovascular function are at higher risk of thermic injury and illness (Belmin, 2003; Green et al., 2006; Kenny et al., 2010; Lundgren et al., 2013) and this may be compounded by the effects of an urban heat island (Mohammad Harmay & Choi, 2023).

Heat stroke is generally more severe than EIHS and can lead to death and/or cardiovascular disease (Bathini et al., 2020; Marchand & Gin, 2022; Michelozzi et al., 2005, 2009; Semenza et al., 1996). The consequences of heat stroke on the cardiovascular system are well‐known and include arrhythmias, conduction disturbances, and myocardial ischemia/injury (Akhtar et al., 1993; al‐Harthi et al., 1992; Hausfater et al., 2010; Marchand & Gin, 2022; Mimish, 2012). However, the effects of EIHS on the cardiovascular system are largely unknown, despite its far more frequent occurrence (Abdelmoety et al., 2018; Harduar Morano & Watkins, 2017). Limited insight regarding the cardiovascular responses to heat stress has been gained in humans (Crandall, 2000; Crandall & Gonzalez‐Alonso, 2010; Crandall & Wilson, 2015; Rowell et al., 1969); however, these investigations generally used mild heating protocols with brief durations that are unlikely to accurately model a naturally occurring EIHS event. Nevertheless, a previous EIHS exposure increases the likelihood of a future cardiac event (Wang et al., 2019), suggesting strongly that EIHS causes lasting damage to the myocardium. A porcine EIHS model allows us to probe questions about the consequences of EIHS in a human‐like model system using temperatures and durations that are more representative of EIHS conditions. Using a porcine EIHS model, we previously discovered EIHS altered cardiac dimensions such that heart weight and length decreased, left ventricle (LV) thickness increased, and right ventricle (RV) thickness decreased after a 24 h exposure (Roths, Freestone, et al., 2023). Given changes in LV and RV thickness and the short period of environmental exposure, we anticipated edema in the LV and decreased water content in RV; however, we were surprised by contrary findings (Roths, Freestone, et al., 2023). Further, we discovered EIHS caused ventricle‐specific changes such that RV had metabolic dysregulation and increased mitochondrial injury, whereas LV did not. Considering the aforesaid, the purpose of this investigation was to determine the extent to which EIHS caused increased proteolysis and altered calcium homeostasis in the LV and RV. We hypothesized that EIHS would increase proteolysis in the RV, whereas in LV, EIHS would cause calcium dysregulation.

## 2 MATERIALS AND METHODS

### 2.1 Animal treatment and experimental design

All animal work was approved by the Institutional Animal Care and Use Committee (IACUC‐18‐314) at Iowa State University. A detailed description of our approach has been previously published (Roths, Freestone, et al., 2023). Briefly, following a 5‐days acclimatization period, 3‐month‐old female pigs were assigned to thermoneutral (TN; 20 ± 0.2°C; *n* = 8) or EIHS (37.4 ± 0.2°C; *n* = 8) conditions for 24 h. All pigs were given ad libitum access to feed and water. As previously reported, all pigs were fed a standard diet formulated to meet or exceed the nutritional requirements of growing pigs (Mayorga, Freestone, et al., 2024). Upon completion, pigs were euthanized with captive bolt and exsanguinated, and hearts were collected. Portions of the anterior‐facing LV free‐wall and the anterior RV free‐wall were removed, frozen in liquid nitrogen, and then stored at −80°C until further analysis.

### 2.2 Protein extraction and western blotting

Protein extraction was performed as previously described (Roths, Abeyta, et al., 2023; Roths et al., 2024; Roths, Freestone, et al., 2023). Briefly, LV and RV were powdered on dry ice and protein was extracted from 25 to 40 mg of tissue using a 1:10 weight to volume ratio of whole muscle extraction buffer (10 mM sodium phosphate buffer, pH 7.0, 2% SDS, 1% Halt protease inhibitor single use cocktail, ThermoFischer Scientific #78425). Samples were centrifuged for 15 min at 10,621 ***g*** at 4°C and supernatant was collected. Western blots were performed as previously described (Roths, Abeyta, et al., 2023; Roths et al., 2024; Roths, Freestone, et al., 2023). Briefly, total protein concentrations were measured using Pierce BCA Protein Assay Kit (#23227 ThermoFischer Scientific, USA) per manufacturer's instructions. Samples were diluted in 4× LDS (lithium dodecyl sulfate pH 8.4) Sample Buffer (M00676, GenScript) and denatured at 85°C for 5 min. After heating, 7 μL of each sample (28 μg total protein) were loaded onto 4%–20% SurePage Bis‐Tris gels (M00657, GenScript; 45 min at 180 V) and transferred onto nitrocellulose membranes (#1620112, Bio‐Ra; 1 h at 100 V at 4°C). Objective quantification of Ponceaus S stain was used to verify equal loading as previously described (Roths, Abeyta, et al., 2023; Roths, Freestone, et al., 2023) and membranes were blocked in 5% dehydrated, non‐fat milk in TTBS (50 mmol/L Tris–HCL, 150 mmol/L NaCl, 0.1% Tween20, pH 7.4) for 1 h and probed with primary antibodies (Table 1) overnight at 4°C. Membranes were washed three times for 10 min in TTBS, incubated for 1 h with secondary antibody (anti‐rabbit (CST #7074) or anti‐mouse (CST #7076)) at room temperature, and washed again three times for 10 min in TTBS. Enhanced chemiluminescence (ECL) (#1705062, BioRad, USA) was applied to membranes for 5 min at room temperature and proteins were detected using an Azure Biosystems c600 imaging system and bands were objectively quantified using Azurespot software.

**TABLE 1:** Antibodies and dilutions used for western blot analysis.

Antibody	Company/product No.	Primary dilution	Secondary dilution
Calmodulin (CaM)	Abcam, ab45689	1:1000	1:2000
Calmodulin‐dependent protein kinase II (CaMKII)	Santa Cruz Technologies (SC), #5306	1:500	1:1000
Phophorylated‐Calmodulin‐dependent protein kinase II (p‐CaMKII) (Thr286)	Cell Signaling Techology (CST), #12716	1:500	1:1000
Calpain 1 Large Subunit (Mu‐Type	CST, #2556	1:1000	1:2000
Calpain 2 Large Subunit (M‐Type)	CST, #2539	1:1000	1:2000
Calpastatin	CST, #4146	1:1000	1:2000
Troponin T	Millipore Sigma, #T6277	1:1000	1:2000
Calsequestrin	Invitrogen, #VIIID12	1:500	1:1000
Calumenin	SC, #271357	1:1000	1:2000
Junctophilin2	SC, #377086	1:1000	1:2000
Muscle atrophy F‐box protein (MAFbx/Atrogin‐1)	SC, #166806	1:1000	1:2000
Muscle RING finger protein 1 (MuRF‐1)	SC, #398608	1:1000	1:2000
ORAI1	ProteinTech, #66223–1	1:1000	1:3000
Plasma membrane calcium ATPase (PMCA)	SC, #211917	1:1000	1:2000 mouse
Phospholamban (PLB)	Novus, #NBP2‐19807	1:3000 (5% milk)	1:3000 (5% milk)
Phosphorylated‐Phospholamban (S16/Thr17)	CST, #8496	1:1000	1:2000
Phosphorylated Protein Kinase C (p‐PKC)	Novus Biologicals, #BP2‐19807	1:3000 (5% milk)	1:3000 (5% milk)
Phosphorylated Protein Kinase C (p‐PKC)	CST, #9580	1:1000 (5% milk)	1:4000 (5% milk)
Ryanodine Receptor 2 (RyR2)	Invitrogen, #MA3‐916	1:1000	1:2000
Phospho‐Ryanodine Receptor (p‐RyR2)	Invitrogen, #PA5‐36758	1:1000	1:2000
Sarco/endoplasmic Reticulum Calcium ATPase (ATP2A2/SERCA2)	CST, #4388	1:1000 (5% milk)	1:4000 (5% milk)
Sodium/Calcium exchanger 1 (NXC1)	ProteinTech, #28447	1:1000	1:2000
Stromal interaction molecule 1 (STIM1)	CST, #4916	1:1000	1:2000
Ubiquitin	CST, #3933	1:1000	1:2000
Voltage dependent anion channel 2 (VDAC2)	CST, #9412	1:1000	1:2000

### 2.3 Proteasome activity

For proteasome activity, 25–30 mg of tissue was homogenized in a 1:10 weight to volume ratio in HEPES buffer, centrifuged at 10,000 ***g*** for 30 min at 4°C, and the supernatant collected. Protein concentrations were measured using the Pierce BCA Protein Assay Kit (#23227 ThermoFischer Scientific, USA) per the manufacturer's instructions. Proteasome activity was measured using a 20S Proteasome Assay Kit (APT280, Millipore Sigma) according to manufacturer's instructions.

### 2.4 Statistics

Data were compared using an unpaired two‐tailed *t*‐test with GraphPad Prism 9.3.0 statistical software. Data are presented as means ± standard deviation (SD). Data points two standard deviations away from the mean were considered outliers and were excluded regardless of group or direction. Significance was established as *p* < 0.05.

## 3 RESULTS

**FIGURE 1:**
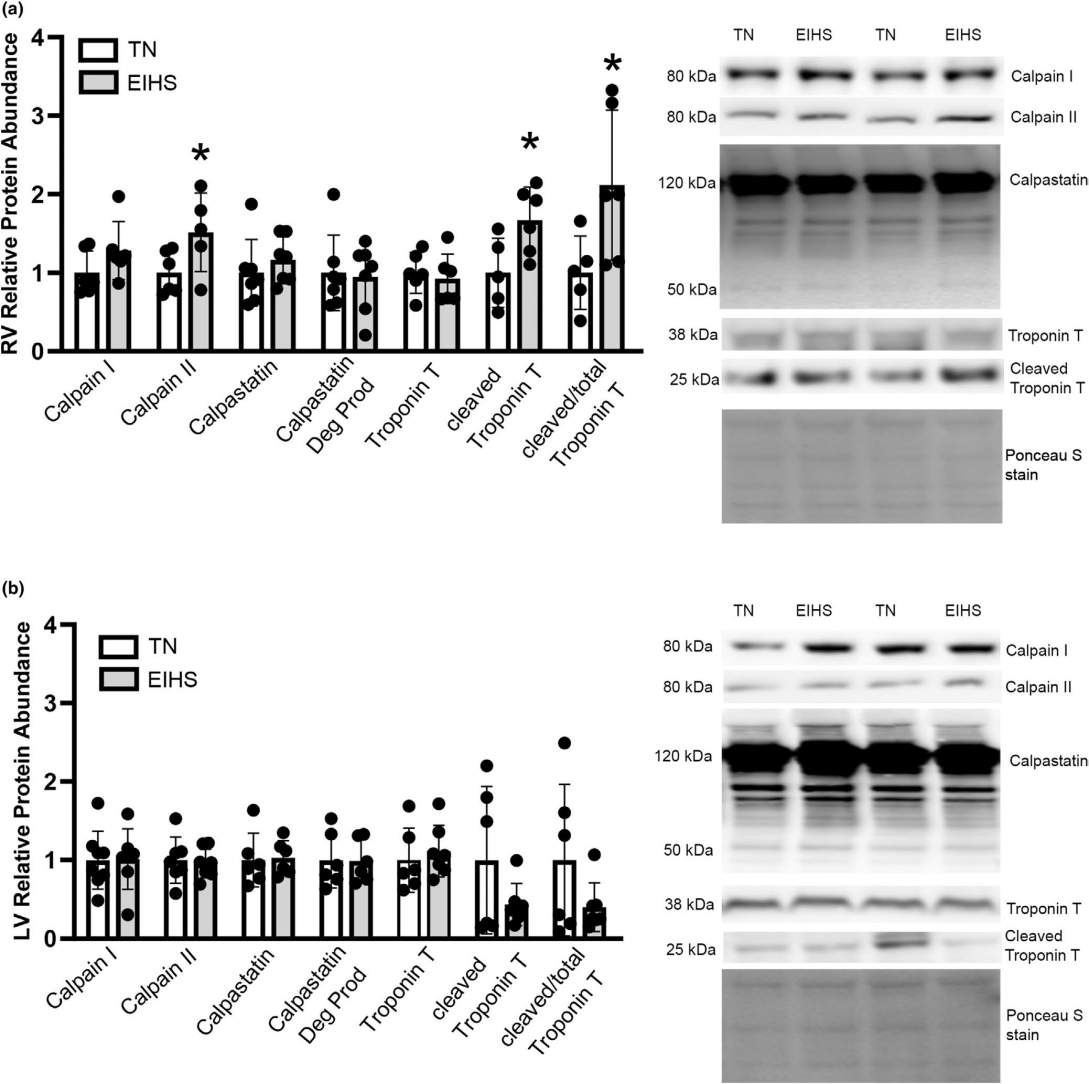
Environment‐induced heat stress (EIHS) effects on proteolysis markers. In RV (a) relative protein abundance of Calpain II, cleaved Troponin T, and cleaved Troponin T/total Troponin T was increased by EIHS compared to TN. Calpain I, calpastatin, calpastatin degradation products, and total Troponin T were similar between groups (a). In LV (b) markers of proteolysis were similar between groups. Treatments: EIHS, environment‐induced heat stress; TN, thermoneutral. Results are expressed as means ± standard deviation. * indicates *p* < 0.05. (a) RV: Calpastatin *n* = 7/group, Calpain I, Calpain II, Troponin T, cleaved Troponin T, and cleaved Troponin T/total *n* = 6/group, one outlier removed from TN cleaved Troponin T and one outlier removed from EIHS Calpain II. (b) LV: Calpain I, Calpain II *n* = 8/group, one outlier removed from EIHS Calpain I, one outlier removed from TN Calpain II; Calpastatin *n* = 6/group; Troponin T *n* = 7/group, one outlier removed from TN.

We previously reported EIHS‐mediated changes in thickness in both RV and LV following 24 h of HS that were independent of water content (Roths, Freestone, et al., 2023), such that the RV was thinner and the LV was thicker following EIHS. To further investigate the cause of decreased RV thickness, we measured markers of proteolysis. In both the RV and LV, relative protein abundance of Calpain‐I was similar between groups (Figure 1a,b). In the RV, relative protein abundance of Calpain‐II was increased by 51% (*p* = 0.05) in EIHS compared to TN (Figure 1a) but was similar in the LV (Figure 1b). Relative protein abundance of calpastatin and degradation products of calpastatin were similar between groups in both the LV and RV (Figure 1a,b). Troponin T is a client protein of calpains (Goll et al., 2003) and the degradation of troponin can be used as an indicator of calpain activity. In the RV, total troponin was similar between groups, but cleaved troponin and the ratio of cleaved troponin/total troponin were increased by EIHS by 62% (*p* = 0.02) and 111% (*p* = 0.04), respectively, compared to TN (Figure 1a). In the LV, relative protein abundance of total troponin, cleaved troponin, and the ratio of cleaved troponin/total troponin was similar between groups (Figure 1b).

**FIGURE 2:**
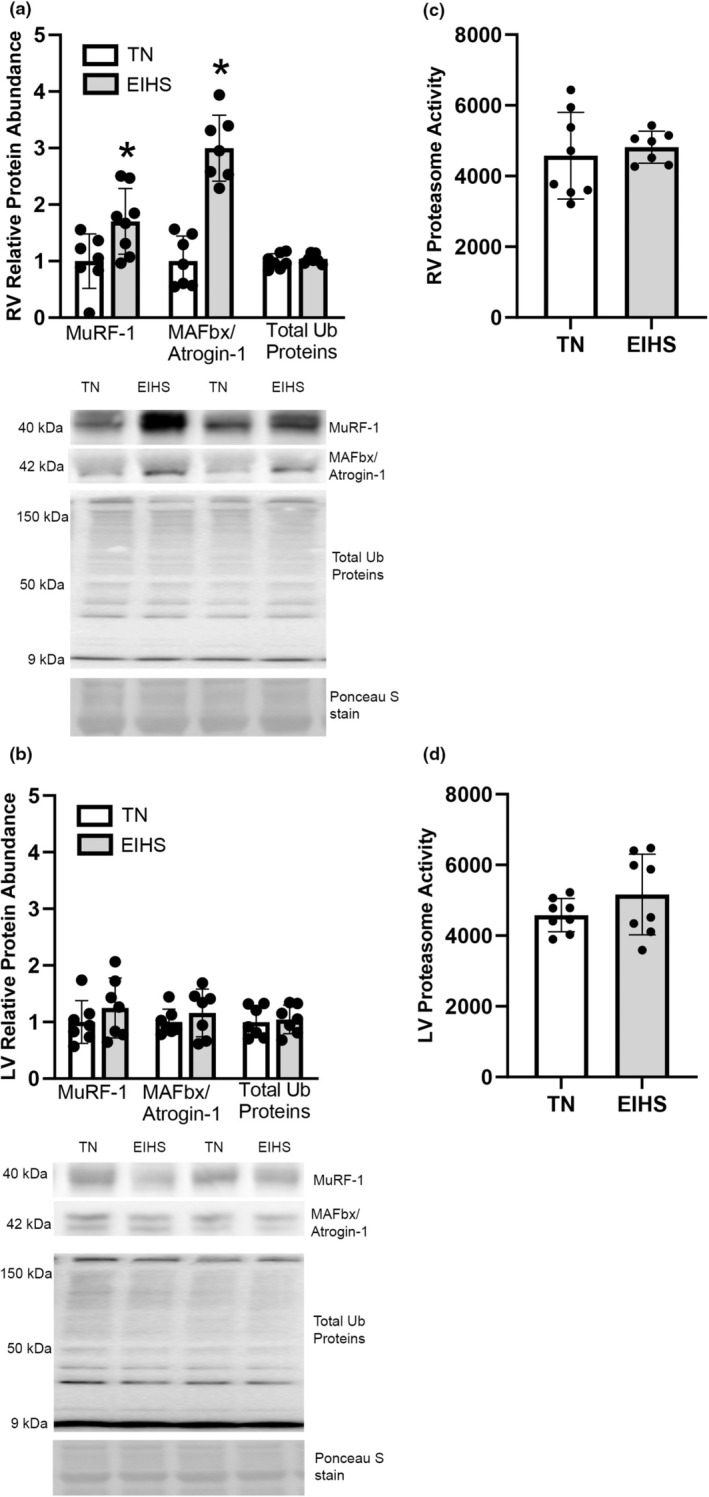
Environment‐induced heat stress (EIHS) effects on E3 ligases and Ubiquitin. In RV (a) relative protein abundance of MuRF‐1 and MAFbx/Atrongin‐1 was increased by EIHS compared to TN, but total ubiquitinated proteins were similar between groups. In LV (b) relative protein abundance of MuRF‐1, MAFbx/Atrongin‐1, and total ubiquitinated proteins was similar between groups. Proteasome activity was similar between groups in RV (c) and LV (d). Treatments: EIHS, environment‐induced heat stress; TN, thermoneutral. Results are expressed as means ± standard deviation. * indicates *p* < 0.05. (a) RV: MuRF‐1 *n* = 8/group, one outlier removed from TN; MAFbx/Atrongin‐1 and total ubiquitin proteins *n* = 7/group. (b) LV: *N* = 7/group. (c) RV: *N* = 8/group, one outlier removed from EIHS. (d) LV: *N* = 8/group.

Calpain products are frequently tagged with ubiquitin via the E3 ligases muscle atrophy F‐box protein (MAFbx/Atrogin‐1) and muscle RING finger protein 1 (MuRF‐1) and routed to the proteasome for further degradation (Adams et al., 2007; Bell et al., 2016; Goll et al., 2003; Kedar et al., 2004; Smuder et al., 2010). In the RV, EIHS increased relative protein abundance of MuRF‐1 by 70% (*p* = 0.02) and MAFbx/Atrogin1 by 199% (*p* < 0.01) compared to TN (Figure 2a), but in the LV, MuRF‐1 and MAFbx/Atrogin‐1 were similar between groups (Figure 2b). Additionally, in both the RV and LV, the relative protein abundance of total ubiquitinated proteins was similar between groups (Figure 2a,b). Proteasome activity for the RV and LV was similar between groups (Figure 2c,d).

**FIGURE 3:**
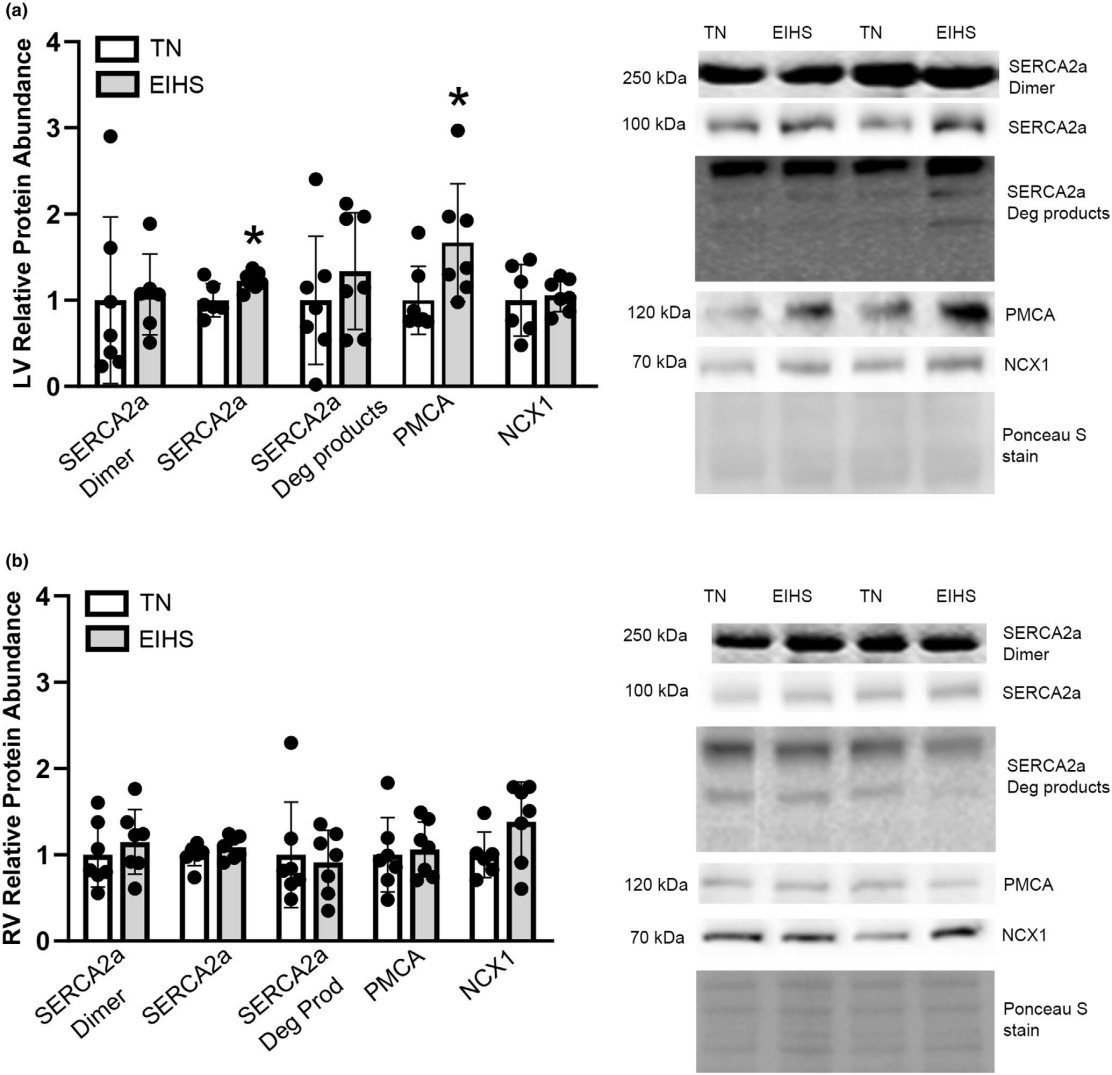
Calcium pumps were increased by EIHS in LV. In LV (a) relative protein abundance of SERCA2a and PMCA were increased by EIHS compared to TN but were similar between groups in RV (b). Relative protein abundance of NCX1, SERCA2a dimer, and SERCA2a degradation products were similar between groups in both LV (a) and RV (b). Treatments: EIHS, environment‐induced heat stress; TN, thermoneutral. Results are expressed as means ± standard deviation. * indicates *p* < 0.05. (a) LV: TN *n* = 7/group, EIHS *n* = 7/group, one outlier removed from TN SERCA2a and NCX1, one outlier removed from EIHS SERCA2a dimer. (b) RV: TN *n* = 7/group, EIHS *n* = 7/group, one outlier removed from TN NCX1.

As ventricular thickness could also be impacted by resting tonicity, we considered proteins involved in the maintenance of calcium homeostasis (Aureliano et al., 2022; Brini & Carafoli, 2011). In the LV, relative protein abundance of sarco(endo)plasmic reticulum calcium ATPase (SERCA)2a, which pumps calcium into the SR, was increased by 18% (*p* = 0.02) by EIHS compared to TN (Figure 3a), but was similar between groups in the RV (Figure 3b). In both the LV and RV, relative protein abundance of SERCA2a dimer and degradation products was similar between groups (Figure 3a/B). Relative protein abundance of plasma membrane calcium ATPase (PMCA) was increased by EIHS in the LV by 66% (*p* = 0.04) compared to TN (Figure 3a), whereas in the RV it was similar between groups (Figure 3b). Relative protein abundance of the Na^+^/Ca^2+^ exchanger (NCX) was similar between groups in both the LV and RV (Figure 3a,b).

**FIGURE 4:**
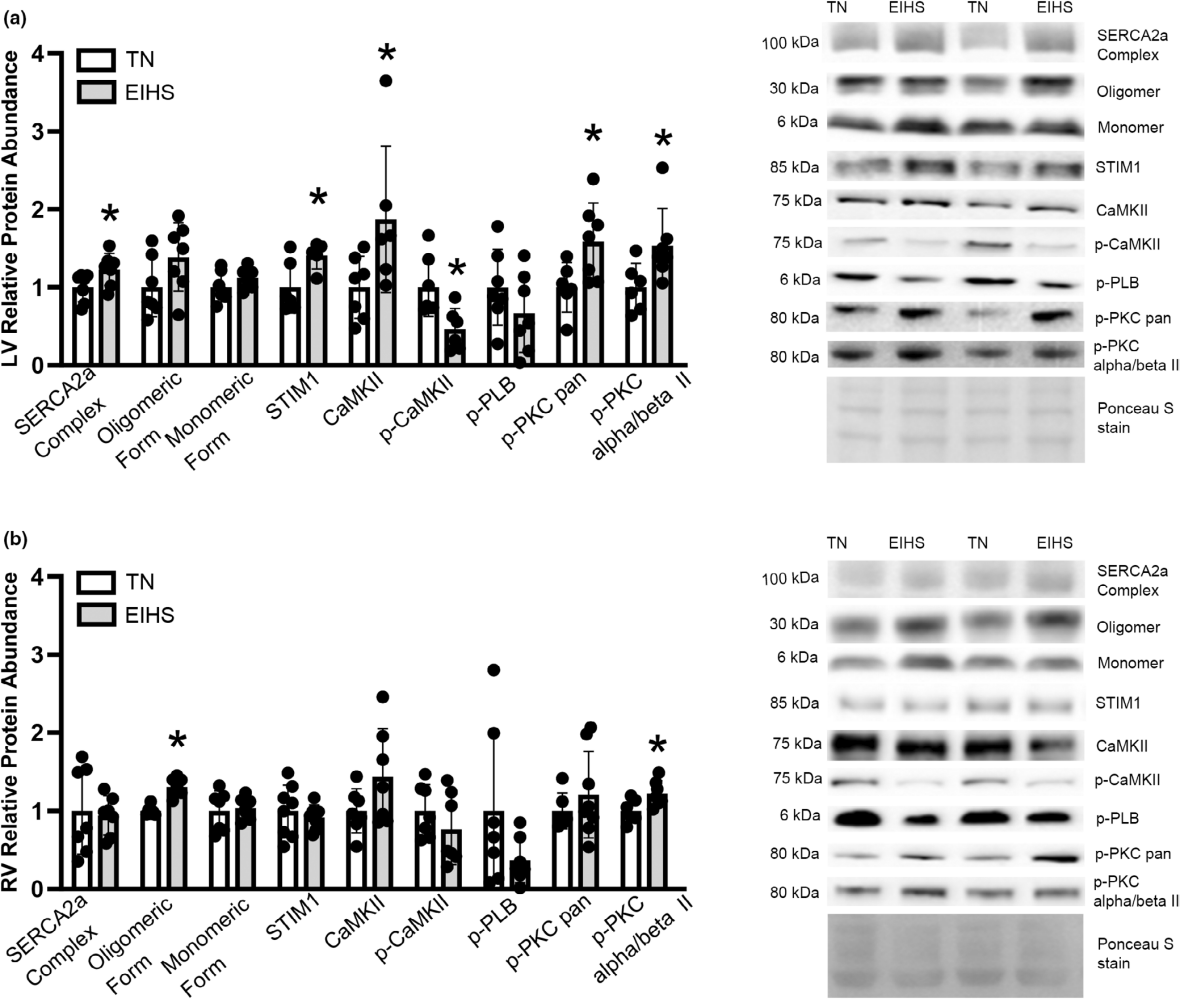
Environment‐induced heat stress altered calcium regulatory proteins in LV. In LV (a) environment‐induced heat stress increased the relative protein abundance of the PLB/SERCA2a complex, STIM1, CaMKII, p‐PKC pan, and p‐PKC alpha/beta II compared to TN; while the monomeric and oligomeric forms of PLB, p‐CaMKII, and p‐PLB were similar between groups. In RV (b) relative protein abundance of the oligomeric form of PLB and p‐PKC alpha/beta II increased by EIHS compared to TN; whereas the monomeric form of PLB, PLB/SERCA2a complex, STIM1, CaMKII, p‐CaMKII, p‐PLB, and p‐PKC pan were similar between groups. Treatments: EIHS, environment‐induced heat stress; TN, thermoneutral. Results are expressed as means ± standard deviation. * indicates *p* < 0.05. (a) LV: Monomer, oligomer, SERCA2a complex, CaMKII, p‐CaMKII, p‐PLB, p‐PKC pan, and p‐PKC alpha/beta II *n* = 7/group, one outlier from EIHS CaMKII and p‐CaMKII and one outlier removed from TN p = PKC pan and p‐PKC alpha/beta II; STIM1 *n* = 6/group, one outlier removed from EIHS. (b) RV: Monomer, oligomer, SERCA2a complex, CaMKII, p‐CaMKII, p‐PLB, *n* = 7/group, one outlier removed from TN oligomer; STIM1 *n* = 8/group, one outlier removed EIHS; p‐PKC pan and p‐PKC alpha/beta II TN *n* = 6/group, EIHS *n* = 8/group, one outlier removed from EIHS p‐PKC alpha/beta II.

In the heart, SERCA2a activity is regulated, in part, by phospholamban (PLB) (Bhupathy et al., 2007). In the LV, relative protein abundance of monomeric and oligomeric PLB was similar between groups (Figure 4a); however, EIHS increased relative protein abundance of the SERCA2a/PLB complex by 18% (*p* = 0.04) compared to TN (Figure 4a). Conversely, in the RV, relative protein abundance of monomeric PLB and the SERCA2a/PLB complex was similar between groups (Figure 4b); however, EIHS increased the oligomeric form by 23% (*p* < 0.01) compared to TN (Figure 4b). Stromal interaction molecule 1 (STIM1) is a transmembrane protein that can maintain SERCA activity in the presence of PLB (Collins et al., 2013; Zhao et al., 2015). In the LV, EIHS increased relative protein abundance of STIM1 by 29% (*p* = 0.03) compared to TN (Figure 4a), but not in the RV (Figure 4b). Furthermore, PLB can be phosphorylated by calcium calmodulin‐dependent protein kinase II (CaMKII) and protein kinase C (PKC) (Mattiazzi & Kranias, 2014). In the LV, EIHS increased CaMKII by 87% (*p* = 0.04) compared to TN (Figure 4a) and p‐CaMKII decreased by 53% (*p =* 0.01); however, p‐PLB was similar between groups (Figure 4a). In the RV, CaMKII, p‐CaMKII, and p‐PLB were similar between groups (Figure 4b). Relative protein abundance of p‐PKC (using a pan antibody) and p‐PKC (α/β II isoforms) were increased by EIHS in the LV by 58% (*p* = 0.03) and 52% (*p* = 0.05), respectively, compared to TN (Figure 4a). In the RV, EIHS increased p‐PKC (alpha/beta II isoforms) by 21% (*p* = 0.02) compared to TN (Figure 4b); however, p‐PKC (pan) was similar between groups (Figure 4b).

**FIGURE 5:**
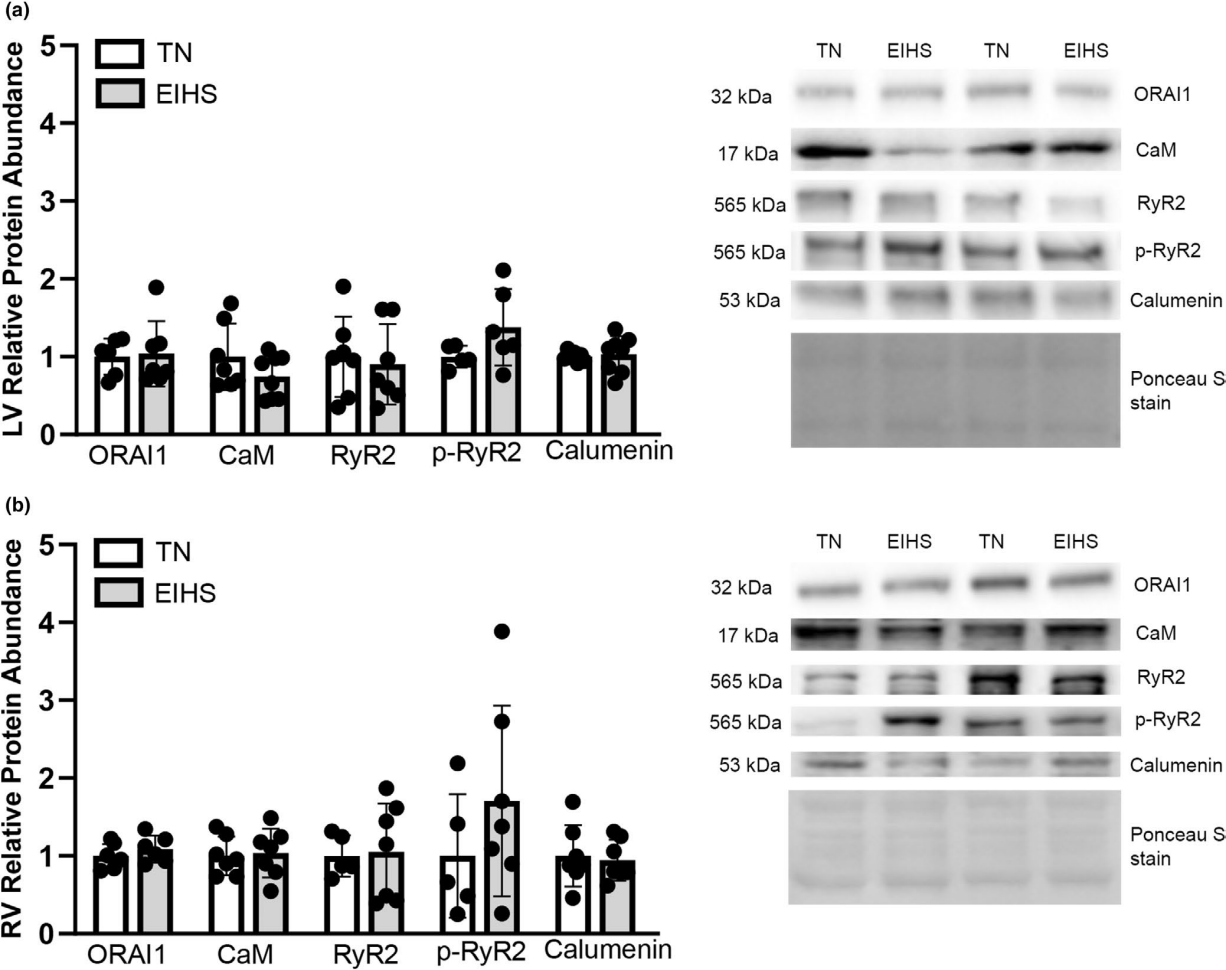
Some calcium regulatory proteins did not change as a result of environment‐induced heat stress (EIHS). In LV (a) and RV (b), relative protein abundance of ORAI1, CaM, RyR2, p‐RyR2, and calumenin was similar between groups. Treatments: EIHS, environment‐induced heat stress; TN, thermoneutral. Results are expressed as means ± standard deviation. * indicates *p* < 0.05. (a) LV: ORAI1 and RyR2 *n* = 7/group, one outlier removed from TN ORAI1; CaM and calumenin, *n* = 8/group, one outlier removed from TN CaM and calumenin; p‐RyR2 *n* = 6/group, one outlier removed from TN. (b) RV: ORAI1, CaM, calumenin *n* = 7/group, one outlier removed from EIHS ORAI1; RyR2 and p‐RyR2 TN *n* = 5/group, EIHS *n* = 7/group.

In both the LV and RV, ORAI1, a part of store‐operated calcium entry, was similar between groups (Figure 5a,b). Calmodulin (CaM) is activated by calcium, and it is involved in the regulation and transduction of calcium signaling (Beghi et al., 2022; Sorensen et al., 2013), ryanodine receptor (RyR2) function, and, given its function, may also regulate the cardiac action potential (Abriel & Kass, 2005; Chou et al., 2015; Kang et al., 2020; Pitt, 2007; Sorensen et al., 2013). In both LV and RV, the relative protein abundance of CaM, RyR2, and p‐RyR2 was similar between groups (Figure 5a,b). Calumenin is a calcium‐binding protein and interacts with SERCA and RyR2 in the SR, supporting a dual role in SR calcium uptake (Sahoo et al., 2009). The relative protein abundance of calumenin was similar between groups in both the LV and RV (Figure 5a,b).

**FIGURE 6:**
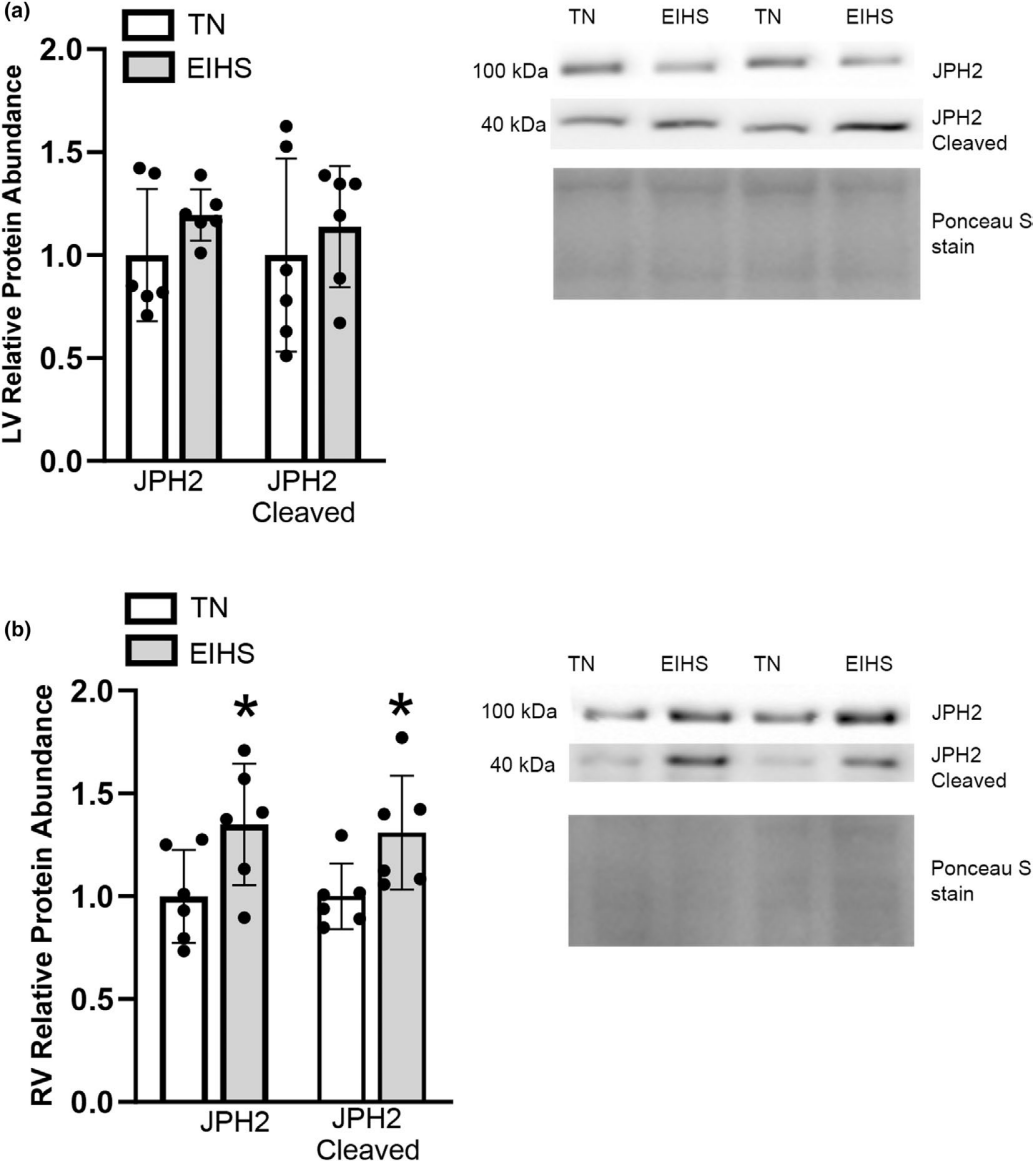
Environment‐induced heat stress (EIHS) effects on Junctophilin2. In LV (a), relative protein abundance of JPH2 and cleaved JPH2 was similar between groups. In RV (b) relative protein abundance of JPH2 and cleaved JPH2 was increased by EIHS compared to TN. Treatments: EIHS, environment‐induced heat stress; TN, thermoneutral. Results are expressed as means ± standard deviation. * indicates *p* < 0.05. (a) LV: TN *n* = 6/group, EIHS *n* = 6/group. (b) RV: TN *n* = 6/group; EIHS *n* = 6/group.

Junctophilin type 2 (JPH2) maintains the dyadic structure between the plasma membrane and SR/ER in the heart (Garbino & Wehrens, 2010), which is important for efficient excitation‐contraction coupling (Garbino & Wehrens, 2010). Furthermore, JPH2 can interact with RyR2 in calcium release and with SERCA in calcium uptake (Jiang et al., 2016; Landstrom et al., 2023; Luo et al., 2022; Quick et al., 2016). In the RV, the relative protein abundance of JPH2 was increased by 34% (*p* = 0.04) in EIHS compared to TN (Figure 6b); however, JPH2 was similar between groups in the LV (Figure 6a). Junctophilin type 2 can be cleaved by calpains and release a product with transcription factor activity (Guo et al., 2018). In the RV, consistent with increased calpain activity, cleaved JPH2 was increased by 30% (*p* = 0.03) (Figure 6b), but in the LV, cleaved JPH2 was similar between groups (Figure 6a).

**FIGURE 7:**
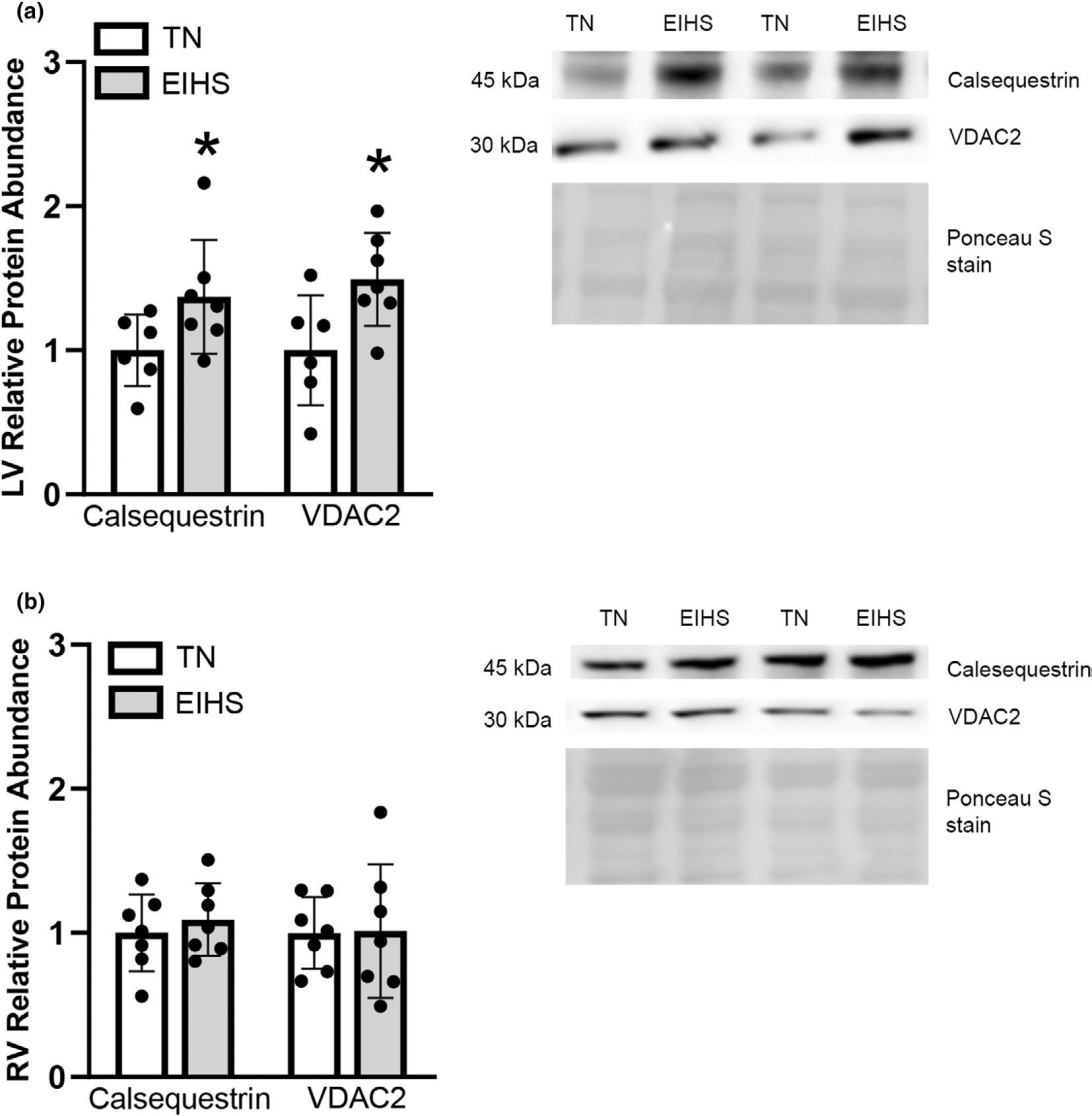
Environment‐induced heat stress (EIHS) increased calsequestrin and VDAC2 in LV. In LV (a), relative protein abundance of calsequestrin and VDAC2 was increased by EIHS compared to TN; but was similar between groups in RV (b). Treatments: EIHS, environment‐induced heat stress; TN, thermoneutral. Results are expressed as means ± standard deviation. * indicates *p* < 0.05. (a) LV: TN *n* = 7/group, one outlier removed from calsequestrin and VDAC2, EIHS *n* = 7/group. (b) RV: TN *n* = 7/group, EIHS *n* = 7/group.

Calsequestrin is a calcium binding protein localized to the SR and serves to regulate calcium uptake and release (Sun et al., 2021). Herein, we discovered the relative protein abundance of calsequestrin was increased by EIHS in the LV by 40% (*p* = 0.03) compared to TN (Figure 7a), but was similar between groups in the RV (Figure 7b). The voltage dependent anion channel (VDAC2) also participates in the maintenance of calcium homeostasis by transporting calcium into the mitochondria (Rosenberg, 2022). Environment‐induced heat stress increased VDAC2 in the LV by 49% (*p* = 0.02) compared to TN (Figure 7a); however, VDAC2 was similar in the RV (Figure 7b).

## 4 DISCUSSION

**FIGURE 8:**
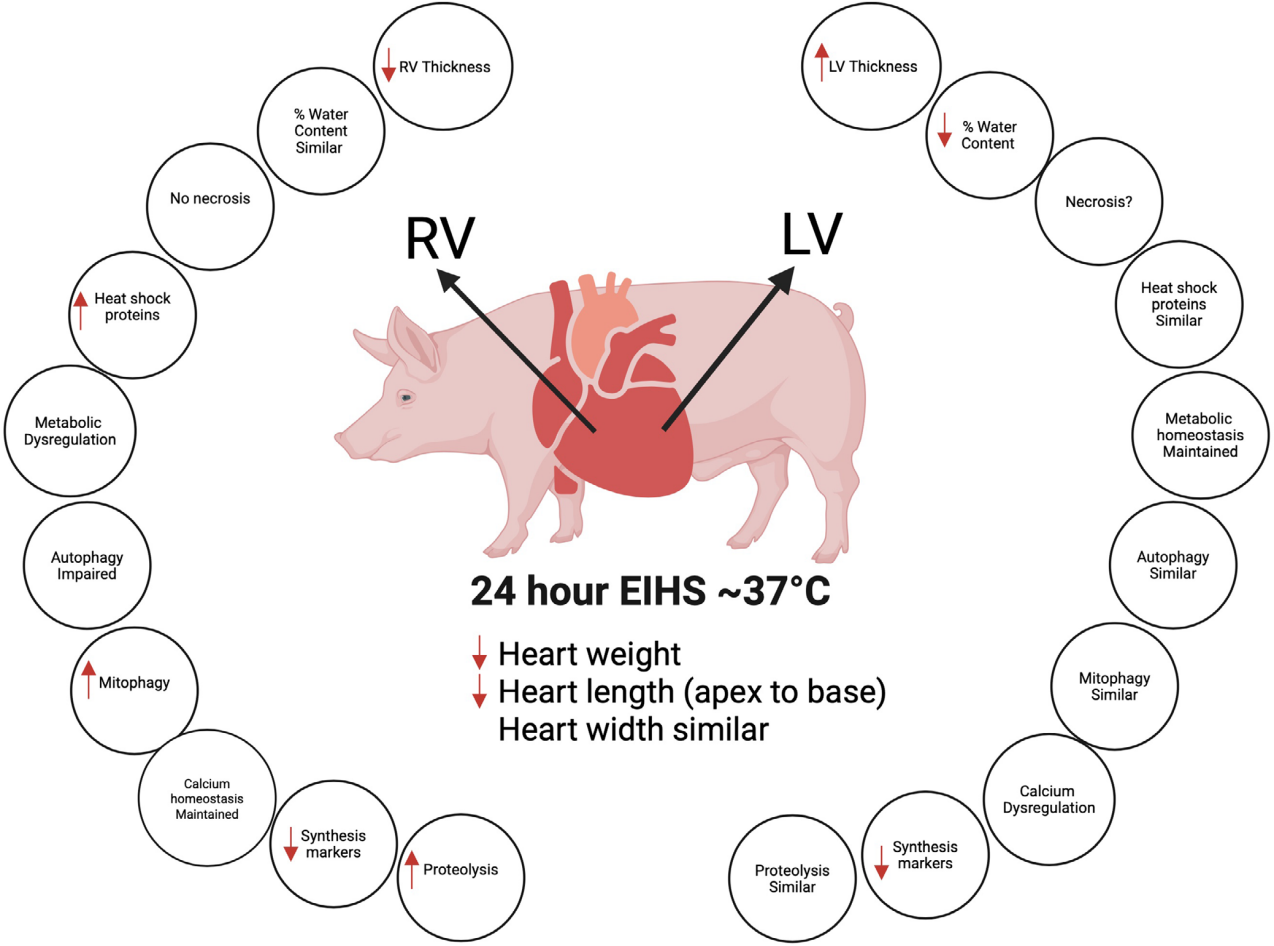
Environment‐induced heat stress causes ventricular‐specific changes. Despite an identical internal environment (i.e., temperature, bathing milleau) 24 h of EIHS caused unique changes in the right (RV) and left ventricle (LV). For example, in the RV, ventricular thickness was decreased and water content was similar, whereas in the LV, ventricular thickness was increased, but water content was decreased. Further, the RV appeared unremarkable following histological inspection, relative abundance of heat shock proteins was increased, and EIHS caused metabolic dysregulation, impaired autophagy, and increased mitophagy. While these changes were not apparent in the LV, in the LV we discovered a trend toward necrosis and calcium dysregulation. Finally, markers of protein synthesis were decreased by EIHS in both RV and LV, and markers of proteolysis were increased in RV but not LV.

The increasing frequency, duration, and intensity of heat events is a current, continuing, and expanding threat to human health. Therefore, gaining an understanding of how EIHS impacts the myocardium is essential to maintain and protect human health. While some insight has been gained by modeling EIHS in humans, these investigations necessarily use relatively brief treatment durations, which prevent accurate recapitulation of natural heating emergencies. A porcine EIHS model overcomes ethical limitations imposed on experiments using humans, has a cardiovascular system that is similar to the human (Lelovas et al., 2014; Tsang et al., 2016), and provides information that may be immediately relevant to agricultural production. Further, an improved understanding of EIHS on cardiac muscle could help identify strategies to prevent injury and develop therapeutic interventions. We have previously discovered EIHS‐mediated changes in thickness in both the RV (thinner) and LV (thicker) following 24 h of EIHS that were independent of water content (Roths, Freestone, et al., 2023) (Figure 8). Given these changes in cardiac structure, we hypothesized that EIHS would increase markers of proteolysis in the RV and indices of calcium dysregulation in the LV.

Calpains are cysteine proteases that are activated in response to increased cytosolic calcium and inhibited by calpastatin (Goll et al., 2003). Calpains frequently degrade sarcomeric and cytoskeletal proteins, among other substrates (Goll et al., 2003). These calpain products can be ubiquitinated by E3 ligases, and the tagged polypeptide is further degraded by the proteasome. Furthermore, these ubiquitin ligases, MuRF‐1 and MAFbx/Atrogin‐1, may also reduce protein synthesis, thus exerting a dual role in regulating cardiac mass (Baskin et al., 2014; Lee & Goldberg, 2011). Given decreased wall thickness in the RV and the prominent role of the calpains and the ubiquitin‐proteasome systems in regulating proteostasis, we expected and discovered increased calpain as well as increased E3 ligases in the RV. Moreover, calpain cleavage of troponin and JPH2 was increased in the RV, which further supports increased calpain activity. Given these outcomes, we were surprised that proteasome activity was not increased in the RV following EIHS when measured directly. When combined with our previously reported indices of impaired synthetic signaling (Roths, Freestone, et al., 2023), the combination of decreased synthesis and increased degradation may be sufficient to support decreased wall thickness in the RV, even with the relatively brief heating duration. Indeed, in skeletal muscle atrophy models, muscle mass is frequently decreased within 24 h (Hanson et al., 2013).

Given the relatively brief environmental treatment (24 h), that edema does not appear to be present, and decreased cardiac length, we reasoned that increased LV thickness could be caused by loss of calcium homeostatic control and subsequent dysfunction of the contraction/relaxation cycle. The SR is an important calcium repository. Release of SR calcium is a key step during excitation‐contraction coupling (ECC) and sequestration of calcium into the SR and cellular calcium efflux are essential components of relaxation. In LV, we discovered broad elevations in calcium‐handling proteins including those associated with the SR (SERCA, calsequestrin), sarcolemma (PMCA), and mitochondria (VDAC2) that collectively would decrease cytosolic calcium. Notably, in the LV, proteins associated with increasing cytoplasmic calcium were similar between groups. In addition to calcium handling proteins, in the LV, EIHS caused changes in several key regulatory proteins. STIM1 interacts with ORAI1 and canonically promotes cellular calcium entry (Collins et al., 2013); however, this interaction may also occur at the SR and promote intracellular calcium clearance (Collins et al., 2013). Likewise, PLB is frequently regarded as a SERCA inhibitor (Bhupathy et al., 2007); however, when phosphorylated (Ser 16) or in the presence of elevated calcium, PLB can facilitate SERCA function (Weber et al., 2021). While p‐PLB was similar between groups, we note that the PLB/SERCA complex was increased as was p‐PKC, which may promote SERCA activity via PLB phosphorylation at Ser 16 (Weber et al., 2021). Hence, in total, these data raise the possibility of EIHS‐mediated loss of calcium homeostatic control, which the cardiomyocytes are working to re‐establish through increased calcium sequestration and clearance. Within that framework, we speculate that elevated intracellular calcium is interfering with contraction/relaxation mechanisms and leaving the LV in a hypercontracted state detected as a shorter cardiac length and increased LV wall thickness and creating a likely diastolic dysfunction. Indeed, such changes in calcium regulation may exacerbate underlying cardiomyopathies and increase the risk of sudden cardiac death during EIHS, notably without the necessity for heat stroke‐provoking environmental conditions (Wang et al., 2019). At least some evidence supporting this position can be found with noted increased mortality attributed to cardiac complications in pigs in the summer months (D'Allaire et al., 1996; Kikuti et al., 2023) and increased likelihood of cardiovascular events following EIHS in humans (Wang et al., 2019). Of interest, despite biochemical dysregulation previously reported in the RV (Roths, Freestone, et al., 2023) and reported herein, RV thickness was decreased by EIHS, and calcium handling and regulatory proteins were largely similar between groups, suggesting maintenance of calcium homeostasis by this ventricle.

There are limitations to this work that should be considered. First, we were not able to measure cardiac function or electrical activity in this investigation. Given apparent changes in calcium handling and subsequent dysregulation of the contraction/relaxation cycle, there is a likelihood of diastolic and even systolic dysfunction, and we note these as urgent measures for future study. Environment‐induced HS‐mediated changes to electrical activity are also ill‐defined. While biochemical changes detected herein may be predictive of alterations detected via electrocardiogram, only minor changes have been previously reported (Abdelmoety et al., 2018). Notably, these data were collected with the chaos of large‐scale emergency management of thermic injury victims, and follow‐up was not possible. Second, while animals did have ad libitum access to water, we did not directly measure hydration status in these animals. Nevertheless, like in our previous work (Boddicker et al., 2014; Mayorga et al., 2021; Mayorga, Horst, et al., 2024; Rudolph et al., 2024), we found that hematocrit was similar between TN and EIHS animals (Mayorga, Freestone, et al., 2024). Further, in our previous work, changes in sodium and potassium are not obviously suggestive of dehydration (Boddicker et al., 2014; Rudolph et al., 2024). While these outcomes do not apparently support dehydration, we cannot exclude a complicating role of thermic injury on these markers, which may mask dehydration. Moreover, like other component stressors of thermic injury (i.e., hyperthermia, endotoxemia, endocrine changes, etc.), should animals be dehydrated, it may be an unavoidable consequence of naturally occurring EIHS, as animals had ad libitum access to water. Indeed, objective determination of hydration status is important for future management of EIHS victims.

Increased heat exposure continues to be a threat to human and animal health, and our present and previous findings make apparent that EIHS jeopardizes cardiac health. We previously discovered that EIHS changed cardiac structure in a ventricle‐specific manner. Decreased RV thickness may be driven, at least in part, by proteolysis as well as metabolic dysregulation, increased mitochondrial injury, and blunted synthesis, as previously reported (Roths, Freestone, et al., 2023). In contrast, in the LV with increased wall thickness, we did not find evidence of altered proteostasis but rather loss of calcium homeostasis, which could lead to functional impairment via interference with contraction/relaxation cycling in a diastolic dysfunction. These data further emphasize that the RV and LV behave differently in response to the same EIHS event and underscore that they experience different stressors during EIHS or respond differently to the same constellation of stressors that occur during EIHS.

## CONFLICT OF INTEREST STATEMENT

The authors have no conflicts to declare.

## ETHICS STATEMENT

All animal work was approved by the Institutional Animal Care and Use Committee (IACUC‐18‐314) at Iowa State University.
